# Effects of Carotid plaque Crouse score and serum Hcy on the location of white matter hyperintensities

**DOI:** 10.3389/fneur.2025.1533108

**Published:** 2025-04-02

**Authors:** Yue Liu, Xintao Tian, Xinrui Cheng, Chunyan Jia, Cuiping Li, Shaonan Yang

**Affiliations:** ^1^Department of Neurology, The Affiliated Hospital of Qingdao University, Qingdao, Shandong, China; ^2^Department of Urology, The Affiliated Hospital of Qingdao University, Qingdao, Shandong, China; ^3^Department of Critical Care Medicine, The Affiliated Hospital of Qingdao University, Qingdao, Shandong, China

**Keywords:** Crouse score, Hcy, CSVD, WMH, PVWMH, DSWMH

## Abstract

**Background and objective:**

Carotid plaque Crouse score and serum homocysteine (Hcy) are closely associated with white matter hyperintensities (WMH). In recent years, it had been found that the pathological mechanism of periventricular WMH (PVWMH) and deep subcortical WMH (DSWMH) was different. In this study, we aimed to further determine the respective effects of Carotid plaque Crouse score and serum Hcy on the location of WMH.

**Methods:**

We recruited 284 patients with lacunar infarction admitted to the Affiliated Hospital of Qingdao University and conducted a retrospective cohort study. The level of serum Hcy was determined by ELISA. Carotid plaque Crouse score was evaluated by cervical vascular ultrasound. The severity of PVWMH and DSWMH was graded using a manual rating scale. Logistic regression analysis was performed to explore the relationship between Crouse score, serum Hcy and PVWMH and/or DSWMH. The critical point which Crouse score and serum Hcy played a role was determined by Quartile method.

**Results:**

After adjusting for confounding variables, Logistic regression showed that PVWMH was associated with age, hypertension, Hcy and Crouse score; DSWMH was associated with age, hypertension, and Crouse score but not with Hcy. Quartile analysis indicated that Crouse score > 0.39 was associated with the occurrence of PVWMH and DSWMH, while Hcy > 12.48 was only associated with the occurrence of PVWMH.

**Conclusion:**

Crouse score is associated with both PVWMH and DSWMH. High levels of Hcy is associated with the occurrence of PVWMH, but not DSWMH.

## 1 Introduction

White matter hypersignaling (WMH) accounts for 40% of the disease burden of cerebral small vessel diseases (CSVD) and is the most common imaging manifestation of CSVD (1). Its manifestations are mainly high signal in T2-weighted sequence and superior or low signal in T1-weighted sequence in cranial magnetic resonance imaging (MRI) (2). A study showed that the overall prevalence of WMH in young adults was as high as 25%, and this prevalence increased with age (3). WMH is associated with a decline in daily functional abilities, gait and mood disturbances, and may ultimately lead to cognitive decline, dementia, and stroke (4). As the poor prognosis of WMH becomes more evident, there has been increasing interest in understanding its pathological mechanisms and risk factors. According to the location of WMH, it can be divided into periventricular WMH (PVWMH) and deep subcortical WMH (DSWMH) (5). Generally, these two types of WMH usually develop and progress simultaneously. In recent years, clinicians have gradually found that the influencing factors of WMH in different regions of the brain are not the same. Hannawi et al. showed that PVWMH was more susceptible to hemodynamic changes than DSWMH (6). Other studies showed that PVWMH was closely associated with cognitive impairment and ischemic stroke (7). Several prospective studies indicated that DSWMH was related to emotional and gait disorders (8, 9). A recent large-scale genomic study revealed that PVWMH and DSWMH shared unique genetic structures (10). A study found that PVWMH and DSWMH had distinct histopathological features on the brain tissue pathology of 11 elderly patients (11). The pathological features of DSWMH include axonal loss, vacuolization, and arteriosclerosis (12). In contrast, the pathology of PVWMH is characterized by ependymal loss, discontinuous demyelination, and subependymal gliosis (13). These suggest that there are differences in the risk factors and pathogenesis between PVWMH and DSWMH.

In recent years, carotid atherosclerosis (CAS) and endothelial dysfunction have been considered important risk factors for WMH and been widely studied (14–17). CAS can impair intracranial microcirculation (18). The Crouse score is a quantitative index for evaluating the severity of CAS (19). Studies showed that WMH was associated with atherosclerosis (20). Homocysteine (Hcy) is a sulfur-containing amino acid and a precursor in the metabolism of methionine (21–23). Hcy promotes the development of WMH by damaging endothelial cells (24–26). To date, most studies focused on the impact of CAS and Hcy on the severity of WMH, but only a few studies showed the impact of CAS and Hcy on WMH location (27–30). In this study, we aimed to assess the impact of CAS (quantified by the Crouse score) and the level of serum Hcy on PVWMH and DSWMH.

## 2 Subjects and methods

### 2.1 Subjects

In this study, we recruited patients admitted to the Department of Neurology at the Affiliated Hospital of Qingdao University for acute ischemic stroke between September 2022 and April 2024 (study population: Asian population). We conducted a hospital-based retrospective cohort study. A total of 284 subjects were included in the study. All subjects underwent a detailed medical history review, neurological examination, risk factor assessment, and imaging studies. Imaging examinations included brain computed tomography (CT), MRI, and carotid artery ultrasonography. These subjects all conformed to the expert consensus on diagnosis and treatment of CSVD (31). Exclusion criteria included other stroke subtypes, non-vascular WMH, hepatic or renal insufficiency, systemic inflammation, autoimmune diseases, recent use of folic acid or B vitamins and tumors.

### 2.2 Ethics approval

Our study protocol was designed in accordance with the Declaration of Helsinki and approved by the Ethics Committee of the Affiliated Hospital of Qingdao University. Written informed consent was obtained from all subjects or their close relatives before the study.

### 2.3 Data collection

#### 2.3.1 Collection of serum and testing

We collected fasting blood samples (fasting for at least 8 h) from all subjects between 6 a.m. and 8 a.m. Serum was obtained by centrifugation at 3,000 *g* for 15 min at 4°C and then stored at −80°C. Routine biochemistry (including fasting blood glucose, cholesterol, triglyceride, LDL, HDL, uric acid) was tested by biochemical laboratory of Affiliated Hospital of Qingdao University.

#### 2.3.2 Homocysteine (Hcy) colorimetric assay

According to the kit instructions, we used a colorimetric assay kit (Elabscience, Wuhan, China). to measure the serum Hcy concentration.

#### 2.3.3 Imaging assessment

##### 2.3.3.1 Assessment of WMH

**Figure 1:**
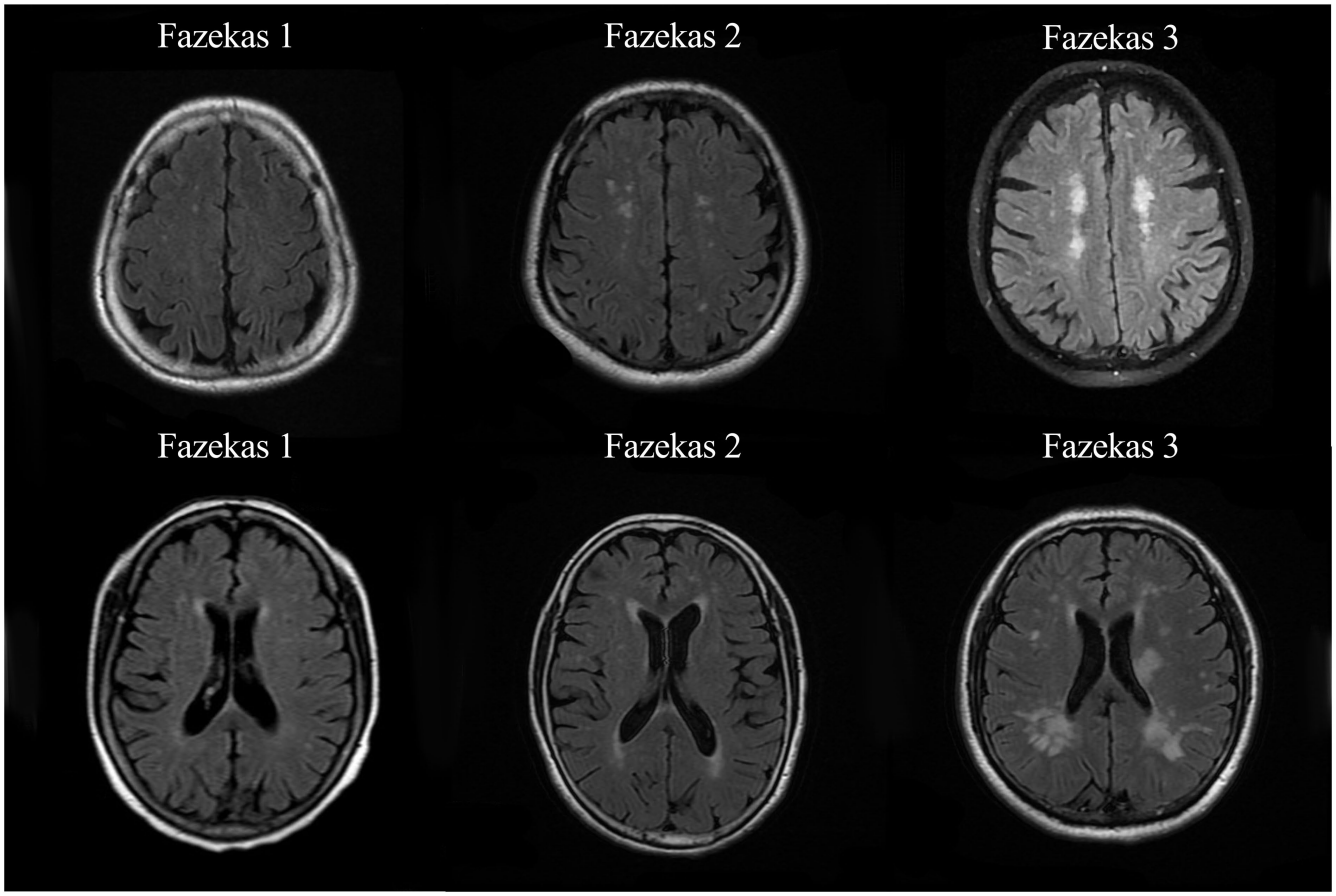
Representative T2-FLAIR images illustrating Fazekas score.

WHM was assessed using the Fazekas scale (32). Definition of PVWMH: White matter areas located around the ventricles of the brain; Definition of DSWMH: Deep white matter areas beneath the cerebral cortex. PVWMH score was as follows: 0, no lesions; 1, Caps or pencil-thin lining around the ventricles; 2, Smooth halo around the ventricles; 3, Irregular periventricular lesions extending into the DSWMH; DSWMH score was as follows: 0, no lesions; 1, punctate foci of lesions; 2, the lesions began to fuse; 3. large confluent areas within the lesions (Figure 1). The Fazekas scale was evaluated by two experienced radiologists, who were not aware of the study. Discrepancies were resolved through discussion to reach a consensus.

##### 2.3.3.2 Carotid plaque Crouse score

Color Doppler ultrasound was used to examine carotid plaques. The patient was placed in a supine position, with the head tilted 45° toward the side opposite the examiner. The examination was conducted gradually from the common carotid artery to the intracranial part of the internal carotid artery. The vertical distance between the intima and the boundary between the media and adventitia in the carotid artery lumen was measured, which was the carotid intima-media thickness (IMT). IMT was recorded in detail, and the presence or absence of plaques was assessed. Carotid artery plaque was defined as a focal IMT ≥ 1.5 mm or a local IMT thickening exceeding (33). Crouse score: The sum of the maximum thicknesses of isolated plaques in the carotid arteries (34).

## 3 Statistical methods

This study is a cross-sectional clinical analysis. SPSS26.0 software was used for statistical analysis. Categorical variables were expressed as frequency (percentage), and differences between groups were compared using the chi-square test. For quantitative variables with a normal distribution, the mean ± standard deviation (x ± s) was be used, and one-way ANOVA was applied for comparisons between groups. Quantitative variables were expressed as M (Q25, Q75) if not normally distributed, with comparisons made using the Mann–Whitney U test. Multivariate logistic regression was conducted to analyze risk factors, calculating the OR and 95% CI. Bilateral *p* < 0.05 indicated statistical significance.

## 4 Results

### 4.1 Comparison of clinical data of WMH patients with different locations of WMH

To investigate the risk factors for PVWMH and DSWMH, 284 subjects were included in this study. The demographic characteristics of the participants were summarized (Table 1). When categorized by PVWMH severity, 212 subjects (72.7%) were classified into the none to mild group, while 72 participants (27.3%) were classified into the moderate to severe group. When categorized by DSWMH severity, 216 participants (76.1%) were classified into the none to mild, while 68 participants (23.9%) were classified into the moderate to severe group. The results showed that there were statistically significant differences in age, smoking history, diabetes, TC, LDL, Hcy and Crouse score between the none to mild PVWMH group and the moderate to severe PVWMH group (*p* < 0.05). Similar significant differences (p < 0.05) were observed between the none to mild and moderate to severe DSWMH groups for the same variables, including age, smoking history, diabetes, TC, LDL, Hcy, and Crouse score. There were statistically significant differences in age, smoking history, diabetes, TC, LDL, Hcy, and Crouse score between none to mild DSWMH group and moderate to severe DSWMH group (*p* < 0.05).

**Table 1:** Clinical characteristics of subjects (*n* = 284) according to the location of cerebral WMH.

	PVWMH		*p* value	DSWMH		*p* value
	None or mild (*n* = 212)	Moderate to severe (*n* = 72)		None or mild (*n* = 216)	Moderate to severe (*n* = 68)	
Male	98 (42.6%)	38 (52.8%)	0.343	102 (47.2%)	34 (50%)	0.781
Age	63 (56.25, 71)	72.10 ± 9.23	0.000*	63 (56.25, 71)	71.57 ± 9.59	0.000*
Smoking	63 (56.25, 71)	72.10 ± 9.23	0.000*	63 (56.25, 71)	71.57 ± 9.59	0.000*
Alcoholism	31 (14.6%)	11 (15.3%)	0.892	33 (15.3%)	9 (13.2%)	0.423
Hypertension	31 (14.6%)	8 (11.1%)	0.545	33 (15.3%)	6 (8.8%)	0.227
Diabetes	38 (50.9%)	52 (72.2%)	0.002*	111 (51.4%)	49 (72.1%)	0.003*
Coronary heart disease	51 (24.1%)	23 (31.9%)	0.214	58 (26.9%)	16 (23.5%)	0.354
Cholesterol	4.53 ± 1.08	4.07 (3.18, 4.79)	0.001*	4.51 ± 1.09	4.19 (3.27, 4.86)	0.004*
Triglyceride	1.14 (0.96, 1.45)	1.26 (0.90, 1.86)	0.214	1.14 (0.96, 1.56)	1.24 (0.91, 1.68)	0.533
Low-density lipoprotein	2.64 ± 0.81	2.3 (1.76, 2.77)	0.002*	2.62 ± 0.82	2.30 (1.84, 2.71)	0.005*
Hyper-density lipoprotein	1.21 ± 0.23	1.17 ± 0.25	0.408	1.21 ± 0.22	1.21 ± 0.25	0.456
Uric acid	301.68 ± 79.04	307.93 ± 80.14	0.563	303.70 ± 80.36	301.87 ± 76.06	0.868
Hcy	10 (9.07, 11.48)	12.14 (9.95, 15.04)	0.000*	10.80 ± 2.87	11.38 (9.68, 14.08)	0.001*
Crouse score	0 (0, 0.26)	0.4 (0.22, 0.53)	0.000*	0 (0, 0.29)	0.4 (0.21, 0.54)	0.000*

Normally distributed data are presented as mean ± standard deviation, and non-normally distributed data are described as median and interquartile range M (P25, P75), *p < 0.05.

### 4.2 Logistic regression analysis of subjects in the moderate to severe PVWMH and DSWMH groups

Logistic regression was performed to explore independent risk factors for PVWMH and DSWMH. The results of Binary Logistic regression analysis for moderate to severe PVWMH and DSWMH groups were as follows (Table 2). The results showed that the variables independently associated with moderate to severe PVWMH included age, hypertension, Hcy, and Crouse score; variables independently associated with moderate to severe DSWMH were age, hypertension, and Crouse score. In univariate analysis, serum Hcy level in moderate to severe DSWMH group was significantly higher than that in the none to mild DSWMH group. But after adjusting for confounders, Hcy was not associated with DSWMH. In contrast, Crouse score was associated with both PVWMH and DSWMH.

**Table 2:** Logistic regression analysis of subjects with moderate to severe PVWMH and DSWMH.

	Moderate to severe PVWMH		Moderate to severe DSWMH	
	OR (95%CI)	*p*	OR (95%CI)	*p*
Age	1.004–1.082	0.010*	1.006–1.080	0.021*
Hypertension	1.198–5.145	0.028*	1.087–4.167	0.027*
Cholesterol	0.610–1.698	0.974	0.617–1.658	0.963
Low-density lipoprotein	0.367–1.340	0.318	0.416–1.438	0.416
Hcy	1.081–1.337	0.002*	0.971–1.164	0.187
Crouse score	6.060–129.742	0.000*	6.081–113.962	0.000*

*p < 0.05.

### 4.3 Quartile assessment of Crouse score and the prevalence of moderate to severe PVWMH and DSWMH

To further evaluate the impact of Crouse score on PVWMH and DSWMH, as well as to evaluate the critical point at which Crouse score was significantly associated with WMH, we divided the Crouse score into four quartiles. Univariate analysis showed significant differences in age, hypertension, diabetes, Hcy, and the prevalence of moderate to severe PVWMH and DSWMH across the quartiles of Crouse score (Supplementary Table 1). In Logistic regression analysis, the prevalence of moderate to severe PVWMH and DSWMH was independently associated with the highest Crouse score quartile (Q4) after adjusting for vascular risk factors (age, hypertension, Hcy, PVWMH and DSWMH) (Table 3).

**Table 3:** Logistic regression analysis of moderate to severe PVWMH and DSWMH based on Crouse score.

	Moderate to severe PVWMH		Moderate to severe DSWMH	
	OR (95%CI)	*p*	OR (95%CI)	*p*
Q1 (≤0)	Ref	–	Ref	–
Q2 (0.1–0.15)	0.386–10.188	0.373	0.026–2.535	0.241
Q3 (0.16–0.3)	0.666–4.923	0.175	0.665–4.482	0.285
Q4 (>0.39)	1.146–8.752	0.007*	1.324–9.100	0.018*

*p < 0.05.

### 4.4 Quartile assessment of Hcy and the prevalence of moderate to severe PVWMH and DSWMH

To further evaluate the impact of Hcy on PVWMH and DSWMH, as well as to evaluate the critical point at which Hcy was significantly associated with WMH, we divided the Hcy into four quartiles. Univariate analysis showed significant differences in gender, age, hypertension, smoking history, Crouse score, and the prevalence of moderate to severe PVWMH and DSWMH across the Hcy quartiles (Supplementary Table 2). In Logistic regression analysis, the prevalence of moderate to severe PVWMH was independently associated with the highest Hcy quartile (Q4) after adjusting for vascular risk factors (gender, age, smoking, Crouse score, PVWMH and DSWMH). However, there was no association in the prevalence of moderate to severe DSWMH across the Hcy quartiles (Table 4).

**Table 4:** Logistic regression analysis of moderate to severe PVWMH and DSWMH based on serum Hcy quartiles.

	Moderate to severe PVWMH		Moderate to severe DSWMH	
	OR (95%CI)	*p*	OR (95%CI)	*p*
Q1 (<9.44)	Ref	–	Ref	–
Q2 (9.44–10.42)	0.258–2.627	0.743	0.356–3.063	0.938
Q3 (10.43–12.48)	0.593–5.354	0.303	0.174–1.575	0.249
Q4 (>12.48)	1.085–9.529	0.035*	0.390–3.262	0.824

## 5 Discussion

Our study confirmed that Crouse score was associated with both DSWMH and PVWMH, while Hcy was mainly associated with PVWMH. Quartile analysis indicated that Crouse score > 0.39 was associated with the occurrence of PVWMH and DSWMH, while Hcy > 12.48 was only associated with the occurrence of PVWMH.

In recent years, the relationship between cranial MRI technology and histopathology has become increasingly stronger, and their combination provides a more comprehensive and precise understanding of brain lesions. The combined application of these two techniques indicated that WMH in different brain regions may have different pathological mechanisms (11). Specifically, the pathological features of DSWMH include less gliosis but more axonal loss, vacuolation, and arteriosclerosis (12). The current view is that this phenomenon is caused by ischemic disease (35–37). This is mainly because that DSWMH is located in the middle cerebral artery blood supply area with less collateral circulation, which is susceptible to ischemic damage (38). The middle cerebral artery is an important branch of the internal carotid artery. Therefore, when atherosclerosis occurs in the carotid artery, the pressure and flow burden on small perforating arteries of the brain will increase, thus destroying the cerebral microcirculation, resulting in the occurrence of DSWMH (18). The Crouse score is a quantitative index to evaluate the severity of CAS, with higher scores indicating more severe atherosclerosis (19). This study found that the Crouse score was an independent risk factor for DSWMH. Quartile analysis of the Crouse score revealed that higher scores were more strongly associated with DSWMH. In addition to DWMH being considered an ischemic lesions, uneven PVWMH is also mainly caused by ischemic changes (13). This region, located in watershed areas, is particularly vulnerable to hypoperfusion (39). Atherosclerosis can reduce blood flow to this region, leading to ischemic injury (40). This study found that Crouse score was also an independent risk factor for PVWMH. Similarly, quartile analysis showed that higher Crouse score was more strongly associated with PVWMH. Therefore, controlling carotid plaque is important for controlling the progression of WMH.

The mechanisms underlying the formation of caps and smooth PVWMH may be the direct result of loss of the ependyma, discontinuous demyelination, and subependymal gliosis, or the indirect consequence of endothelial dysfunction, ventricular dilation, and cerebrospinal fluid leakage, which is essentially non-ischemic (13, 41–43). Two studies on Alzheimer’s disease found Hcy was associated with PVWMH, and autopsy findings showed partial myelin loss and astrocyte proliferation (30, 44). It may be due to the neurotoxicity of high levels of Hcy and the result of hypomethylation (45). In contrast, Hogervorst et al. found that Hcy was associated with DSWMH in Alzheimer’s disease patients (28). It may be related to previous studies that the volume of DSWMH is associated with a reduction in cerebral blood flow in the hippocampal region (44). In an acute stroke population, Hcy was associated with PVWMH. The authors suggested this may be related to Hcy’s effect on endothelial dysfunction (29). This finding was consistent with the results of our this study. We suggested that this could be a result of Hcy-induced damage to endothelial cells and the ependyma, leading to cerebrospinal fluid leakage (42, 46). Other studies on normal populations had different conclusions (27, 47). The inconsistency in these results may be attributed to differences in clinical characteristics of the patients, regional differences, and varying definitions of WMH. However, the specific mechanisms still require further *in vivo* and *in vitro* investigations. In Supplementary Table 3, we summarize the effects of Hcy on different locations of WMH in various populations.

However, we must acknowledge some limitations in our study. First, it is a single-center study with a relatively small sample size. Therefore, larger multi-center studies are needed to validate our findings. Second, we assessed the severity of WMH using only the Fazekas scale, which may introduce some degree of error in the visual assessment and could impact the experimental outcomes. Third, this is a retrospective study, which may be subject to selection bias or confounding bias. Fourth, our study sample only included an Asian population, which may limit the generalizability of the study.

## 6 Conclusion

This study found that Crouse score was associated with both PVWMH and DWMH, while Hcy only was associated with PVWMH. Through Quartile analysis, we identified the threshold values at which the Crouse score and Hcy exerted their effects. This study provided a theoretical basis for determining the timing and targets of intervention.

## Data Availability

The raw data supporting the conclusions of this article will be made available by the authors without undue reservation.
