# Supplementary material for: Effects of Carotid plaque Crouse score and serum Hcy on the location of white matter hyperintensities
Source: Front Neurol. 2025 Apr 2;16:1533108. doi: 10.3389/fneur.2025.1533108 (PMC12000053; doi:10.3389/fneur.2025.1533108)
Supplement: Supplementary file 1 [file Table_1.pdf]

Supplementary materials

Supplementary Table 1. Clinical characteristics of subjects based on serum Crouse score quartile

Crouse score	≤0	0.1～0.15	0.16～0.39	＞0.39	p value
Male	54(43.5%)	6(31.6%)	35(47.9%)	41(60.3%)	0.067
Age	61.94±8.32	66.79±11.45	66.48±9.34	72.00±9.34	0.000*
Smoking	12（9.7%）	3（15.8%）	12（16.4%）	15（22.1%）	0.133
Alcoholism	13（10.5%）	4（21.1%）	12（16.4%）	10（14.7%）	0.481
Hypertension	59（47.6%）	11（59.7%）	52（71.2%）	38（55.9%）	0.015*
Diabetes	28（22.6%）	6（31.6%）	19（26%）	21（30.9%）	0.593
Coronary heart disease	11（8.9%）	2（10.5%）	16（21.9%）	11（16.2%）	0.074
Cholesterol	4.58±1.10	4.23±0.93	4.34±1.04	4.25±1.24	0.057
Triglyceride	1.21（1.00，1.59）	1.15±0.34	1.11（0.92，1.59）	1.16（0.96，1.74）	0.561
Low-density lipoprotein	2.66±0.80	2.49±0.75	2.45±0.83	2.47±1.02	0.168
Hyper-density lipoprotein	1.25±0.24	1.19±0.14	1.19±0.28	1.16±0.21	0.165
Uric acid	300.30±80.08	293.32±67.81	315.27±81.10	303（242.75，351.5）	0.467
Hcy	9.87（8.91，11.52）	10.76±1.97	10.62（9.49，12.67）	11.19（9.62，13.39）	0.008*
PVWMH	11（8.9%）	3（15.8%）	21（28.8%）	37（54.4%）	0.000*
DSWMH	12(9.7%)	1(5.3%)	20(27.4%)	35(51.5%)	0.000*

Note: Normally distributed data are presented as mean ± standard deviation, and non-normally distributed data are described as median and interquartile range M (P25, P75) * $p < 0.05$

Supplementary Table 2. Clinical characteristics of subjects based on serum Hcy quartile

Hcy	＜9.44	9.44～10.42	10.43～12.48	＞12.48	p value
Male	26（36.6%）	28（39.4%）	31（43.7%）	51（71.8%）	0.000*
Age	63.47±9.14	64.51±9.35	66.52±9.55	71（58,77）	0.009*
Smoking	6（8.5%）	5（7.0%）	9（12.7%）	22（31.0%）	0.000*
Alcoholism	6（8.5%）	7（9.9%）	10（14.1%）	16（22.5%）	0.065
Hypertension	39（54.9%）	33（46.5%）	43（60.6%）	45（63.4%）	0.186
Diabetes	18（25.4%）	16（22.5%）	21（29.6%）	19（26.8%）	0.813
Coronary heart disease	9（12.7%）	8（11.3%）	11（15.5%）	12（16.9%）	0.762
Cholesterol	4.50±1.05	4.57±1.03	4.48（3.62，5.22）	4.13±1.07	0.122
Triglyceride	1.11（0.93，1.54）	1.18（1，1.64）	1.17（0.93,1.60）	1.21（1.02,1.65）	0.702
low-density lipoprotein	2.54±0.77	2.68±0.77	2.53（1.81，3.09）	2.41±0.83	0.416
hyper-density lipoprotein	1.26±0.23	1.20±0.24	1.17±0.19	1.18±0.27	0.377
uric acid	282,89±87.30	305.19±67.91	301.97±74.05	312（268，364）	0.05
Crouse score	0（0，0.32）	0（0，0.38）	0.17（0，0.37）	0.28（0，0.51）	0.008*
PVWMH	11（15.5%）	11（15.5%）	17（23.9%）	33（46.5%）	0.000*
DSWMH	13（18.3%）	14（19.7%）	13（18.3%）	28（39.4）	0.006*

Note: Normally distributed data are presented as mean ± standard deviation, and non-normally distributed data are described as median and interquartile range M (P25, P75) * $p < 0.05$

Supplementary Table 3. The effect of Hcy on WMH in different locations across different populations

Author	Subjects	PVWMH or DSWMH	Mechanisms
Hooshmand, B.[1]	Alzheimer's disease	PVWMH	Neurotoxicity and Hypomethylation of Hcy
Waldemar, G.[2]	Alzheimer's disease	PVWMH	Neurotoxicity and Hypomethylation of Hcy
Hogervorst, E.[3]	Alzheimer's disease	DSWMH	Reduced Cerebral Blood Flow in the Hippocampal Region
Gao, Y.[4]	Ischemic stroke	PVWMH	Endothelial Dysfunction and Cerebrospinal Fluid Extravasation
Lee, K. O.[5]	Healthy population	PVWMH	Endothelial Dysfunction
Sachdev, P.[6]	Healthy males	DSWMH	Promoting Atherosclerosis

1. Hooshmand, B., T. Polvikoski, M. Kivipelto, et al., *Plasma homocysteine, Alzheimer and cerebrovascular pathology: a population-based autopsy study.* Brain, 2013. **136**(Pt 9): p. 2707-16.
2. Waldemar, G., P. Christiansen, H.B. Larsson, et al., *White matter magnetic resonance hyperintensities in dementia of the Alzheimer type: morphological and regional cerebral blood flow correlates.* J Neurol Neurosurg Psychiatry, 1994. **57**(12): p. 1458-65.
3. Hogervorst, E., H.M. Ribeiro, A. Molyneux, et al., *Plasma homocysteine levels, cerebrovascular risk factors, and cerebral white matter changes (leukoaraiosis) in patients with Alzheimer disease.* Arch Neurol, 2002. **59**(5): p. 787-93.
4. Gao, Y., S. Wei, B. Song, et al., *Homocysteine Level Is Associated with White Matter Hyperintensity Locations in Patients with Acute Ischemic Stroke.* PLoS One, 2015. **10**(12): p. e0144431.
5. Lee, K.O., M.H. Woo, D. Chung, et al., *Differential Impact of Plasma Homocysteine Levels on the Periventricular and Subcortical White Matter Hyperintensities on the Brain.* Front Neurol, 2019. **10**: p. 1174.
6. Sachdev, P., R. Parslow, C. Salonikas, et al., *Homocysteine and the brain in midadult life: evidence for an increased risk of leukoaraiosis in men.* Arch Neurol, 2004. **61**(9): p. 1369-76.
